# Integrated Affinity Biosensing Platforms on Screen-Printed Electrodes Electrografted with Diazonium Salts

**DOI:** 10.3390/s18020675

**Published:** 2018-02-24

**Authors:** Paloma Yáñez-Sedeño, Susana Campuzano, José M. Pingarrón

**Affiliations:** Departamento de Química Analítica, Facultad de CC. Químicas, Universidad Complutense de Madrid, E-28040 Madrid, Spain; susanacr@quim.ucm.es (S.C.); pingarro@quim.ucm.es (J.M.P.)

**Keywords:** grafting, screen-printed electrodes, diazonium salts, antibodies, nucleic acid, nanomaterials

## Abstract

Adequate selection of the electrode surface and the strategies for its modification to enable subsequent immobilization of biomolecules and/or nanomaterials integration play a major role in the performance of electrochemical affinity biosensors. Because of the simplicity, rapidity and versatility, electrografting using diazonium salt reduction is among the most currently used functionalization methods to provide the attachment of an organic layer to a conductive substrate. This particular chemistry has demonstrated to be a powerful tool to covalently immobilize in a stable and reproducible way a wide range of biomolecules or nanomaterials onto different electrode surfaces. Considering the great progress and interesting features arisen in the last years, this paper outlines the potential of diazonium chemistry to prepare single or multianalyte electrochemical affinity biosensors on screen-printed electrodes (SPEs) and points out the existing challenges and future directions in this field.

## 1. Introduction

Preparation of electrochemical affinity biosensors by immobilization of appropriate bioreceptors onto an electrode platform implies the selection of the electrode material and the methods for immobilization as key steps to achieve the desired final performance [1,2,3]. Accordingly, electrode surface modification using different physical or chemical strategies to be utilized for further biomolecules immobilization is an extensively studied subject in the literature. Physical adsorption, mainly based on the electrostatic interaction between the biomolecule and the support surface, is a simple and economic method that does not damage the activity of the biological material [4,5]. However, poor reproducibility and low sensitivity due to leaching of the adsorbed component may be observed. Another physical immobilization involves entrapment of biomolecules into a three-dimensional network of a natural or synthetic gel [6,7,8] or conducting polymer [9,10]. However, small size molecules are difficult to immobilize using this approach because they can be filtered from the matrix and, moreover, this simple immobilization method is not adequate for the preparation of affinity biosensors because the affinity binding can be strongly hindered.

As it is well known, an interesting alternative to physical methods is the covalent coupling of biomolecules to activated surfaces containing functional moieties with binding capacity. Using the most common electrode materials, which include carbon in all forms (glassy carbon, graphite, carbon nanotubes or graphene), and gold, fabrication of self-assembled monolayers (SAMs) of thiols on gold [11,12], preparation of electrodes by carboxyl-confined [13], silanized [14] or aldehyde-derivatives [15,16], and functionalization by means of click-chemistry [17] have been widely explored. Furthermore, aryl radicals generated from the electrochemical reduction of diazonium salts [18] have also been used to modify electrode surfaces. The reductive process of such salts results in the formation of aryl centered radicals covalently attached onto electrode surfaces after the spontaneous elimination of dinitrogen [19]. The so-called grafting method has demonstrated to be an excellent strategy to be used for further immobilization of biomolecules because of the simple preparation and versatility. In fact, conductive and semiconductive surfaces can be modified with a wide range of functional groups in aqueous solution at room temperature and without sophisticated equipment. Other advantages are the reproducibility, uniformity and stability of the covalently attached organic layer on the surface, which have made this method a suitable choice to develop a wide range of attractive electrochemical affinity biosensors by immobilizing proteins, antibodies, nucleic acids and anchoring nanomaterials [5,20,21,22,23]. In addition, the ability to create a diazonium-modified surface by application of an appropriate potential scan or previous modification of the biomolecules with aryl diazonium allows the selective functionalization of closely spaced microelectrode surfaces with different molecules leading to the construction of multianalyte biosensors [24,25,26]. Electrografting as a method for surface modification was extensively reviewed by Bélanger and Pinson [27]. More recently, applications in electroanalysis were also reviewed [28].

While the use of carbon surfaces (glassy carbon, screen-printed carbon electrodes or SPCEs, graphite, graphene, carbon nanotubes and diamond) has dominated this field, a wide range of other conducting materials such as metals, silicon and indium tin oxide (ITO) have been also employed for diazonium grafting modification with a second to minutes scale reaction time [29,30]. Moreover, a surface can be functionalized with either one or multiple types of aryldiazonium salts to create single or mixed layers, respectively. Indeed, modification with mixed layers is especially relevant in applications where the interface is required to perform multiple functions, and hence different chemical species should be incorporated into the layer, or when the bioplatform should operate in complex biological fluids and a mixture of antifouling and bio-recognition components are needed [30]. However, it is worth to mention that formation of mixed layers using this chemistry is still in the infancy and some challenges remain to be addressed to precisely control their structure to the level reached using alkanethiol mixed SAMs. For example, the unselective reactivity of two aryldiazonium cations makes it difficult to control the surface composition and the highly reactive radicals can grow both on the bare electrode surface and on an as-deposited layer resulting in multilayer formation.

**Figure 1:**
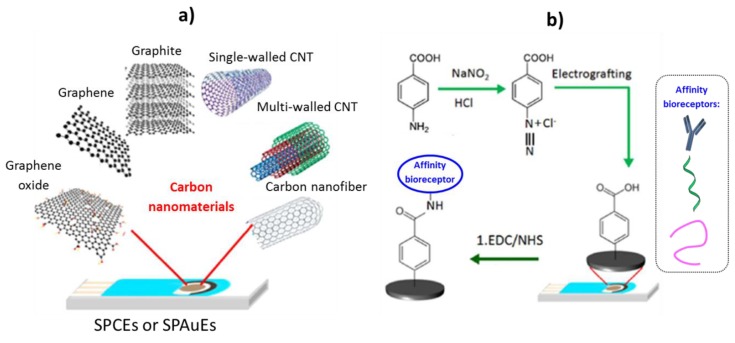
Functionalization of different carbon nanomaterial-modified SPEs (**a**) via diazonium salt reduction and affinity biosensor fabrication (**b**). Reprinted and adapted from [33] with permission.

Regarding grafting onto SPEs, it should be noted that the use of these electrodes is becoming more and more widespread for the preparation of electrochemical affinity biosensors because their disposable nature which simplifies their use, avoids problems with electrode fouling and makes it easier to perform decentralized assays [31,32]. Moreover, the use of screen-printing technology also offers other general interesting advantages such as the utilization of small sample volumes, and the mass production of inexpensive and robust strip solid electrodes suitable to be fabricated in miniaturized sizes, with different materials, in diverse geometries and multiplexed formats [5,33]. Among the different strategies, immobilization of biorecognition components and nanomaterials onto these planar and disposable surfaces pre-functionalized with electrografted diazonium salts has been widely reported in the latest years to develop competitive integrated affinity biosensing platforms (Figure 1). The use of non-disposable electrodes is less attractive for the application of this methodology since once the aryl radical is grafted onto the electrode surface, a regeneration protocol should be implemented according with the employed electrode material for subsequent reutilization. Moreover, one of the main current trends in the development of biosensors is the ability to allow multiplexed detection of several biomarkers with a single sensing platform. However, the selective functionalization of different electrodes remains an obstacle to produce massively multiplexed sensors. In this context, the combination of the screen-printed electrodes ability for multiplexing with the versatility, simplicity, speed and capability offered by electrografting methodology to individually functionalize each working electrode opens up substantial possibilities for the massive fabrication of integrated electroanalytical bioplatforms for the simultaneous determination of biomarkers of the same or different molecular level. These very interesting advantages have led to the fact that in recent years most of electrochemical biosensors configurations using electrodes modified by grafting of diazonium salts have been implemented with screen-printed electrodes.

Considering the aforementioned aspects, this review article discusses the basic concepts of the chemistry involved in aryldiazonium grafting and provides an updated overview of the main strategies reported so far to develop affinity biosensing platforms at SPEs modified with bioreceptors or nanomaterials through representative selected examples. Highlighted examples are classified and discussed in the next sections according to the SPE material and the type of biomolecule immobilized on the electrografted aryldiazonium salt.

## 2. Aryl Diazonium Salt Chemistry in Electrochemical Affinity Biosensing

**Scheme 1:**
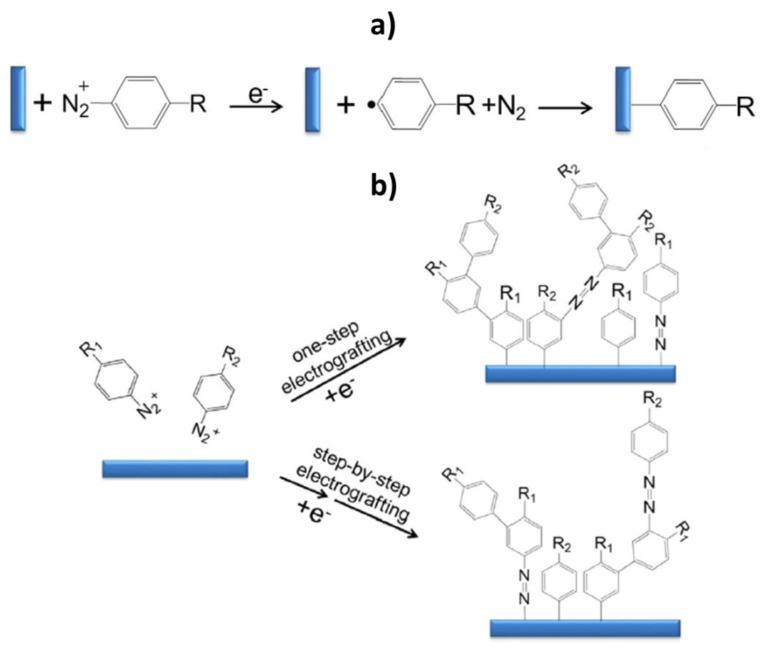
Reaction mechanisms proposed for the preparation of single (**a**) or multiple (**b**) layers onto electrode surfaces through electroreductive electrografting of aryldiazonium salts. In (**b**) R_1_ and R_2_ represent two different substituents. Reprinted from [30] with permission.

Since in 1992 Pinson and co-workers described the reaction mechanism for the modification of carbon electrodes by electrochemical reduction of one or multiple types of aryldiazonium salts (see Scheme 1), this chemistry have demonstrated to be an efficient way to introduce many types of functional groups onto a variety of surfaces (carbon surfaces, metals, silicon and indium tin oxide) with a reaction time scale of seconds to minutes [30].

However, the use of aryl diazonium salt chemistry for the preparation of biosensors may show some drawbacks: (i) the complicated synthesis of new aryl diazonium salts because of the reactivity of the head group; (ii) the fabrication of only single component layers; (iii) the presence of side reactions which compromises surface chemistry control by creating multilayers instead of monolayers; (iv) the need for connection of each electrode to a source of potential for modification; (v) the absence of established methods to apply different chemistries at well-defined surface locations; (vi) when used carbon electrodes, the natural heterogeneity of such surfaces may limit the reproducibility of devices. Despite these problems, the great number of advances made in recent years has provided potential solutions to all these disadvantages and greatly broaden the utility of this powerful surface chemistry for biosensing applications. It is worth to note, for example, that using diazonium salt chemistry, the density of the deposited organic layer on the electrode surface can be controlled by choosing the electrografting protocol and time [20]. Moreover, as indicated in the Introduction section, this chemistry has been used so far for the modification of a variety of conducting surfaces: carbon electrodes (glassy carbon, graphite, screen-printed carbon electrodes, carbon nanotubes [34,35], diamond [36,37,38]), but also metals, silicon and ITO electrodes. In this context, it must be considered that differences existing in the surface chemistry on all these materials have also been investigated.

The in situ formation of aryldiazonium salts has significantly reduced the synthetic burden with no significant differences between the prepared films and the corresponding pre-made salts [39,40]. This is a particularly relevant achievement for the preparation of biosensors since the diazotization step, followed by purification, is a step fraught with problems for some biomolecules. In addition, the great progress made in the preparation of sensing interfaces with mixed layers [30,41,42,43,44] has provided useful insights regarding factors determining the ratio of the two components on the surface compared with the assembly solution [45]. These studies demonstrated, for example, that the concentration of the cation most easily reduced in the electrografted surface is larger than its relative concentration in the mixed solution used for the deposition. In the specific case of electrochemical affinity biosensors, the modification with mixed layers where one component is further modified with bio-recognition species (e.g., antibodies, nucleic acid) and the other component is used to space the attached biorecognition species attached, to immobilize redox species or to minimize nonspecific adsorptions, is particularly relevant [30].

New strategies have been developed for preparing monolayers derived from aryl diazonium salts where the 3- and 5-positions of surface bound diazonium salts are blocked [46] as well as from molecules with a large sacrificial component attached to the 4-position so that, once the layer is formed, is cleaved from the surface leaving a near monolayer. This later was achieved using aryl alkyl hydrazones where hydrolysis of the surface bound molecule leaves a benzaldehyde which is compatible with subsequent coupling of recognition species or a disulfide leaving a surface thiol. Moreover, spontaneous adsorption of aryldiazonium salts and several different strategies which allow simple patterning of the sensing substrate have been reported also on a range of different surfaces [47,48,49,50,51,52]. This is especially important for sensors as it is much more compatible with the bulk manufacture of devices.

Although the application of aryldiazonium salts for the preparation of biosensors was recognized soon after the first report on electrode modification with such derivatives [53], the great explosion of this chemistry for biosensing started ten years ago. Since then, the number of approaches has grown rapidly and has been used for the immobilization of enzymes [54,55,56], antibodies [5,19,20,24,29,33,57,58,59,60,61,62,63,64,65,66,67,68,69,70,71,72,73,74], DNA [24,65,66,67], and PNA probes [68], aptamers [21,69,70], peptides [71,72,73], and even whole cells [74]. Furthermore, the aryldiazonium salt derived layers have demonstrated also very interesting advantages for attaching nanomaterials and other components, to prepare more sophisticated (bio)sensing layers [75,76,77,78,79,80,81,82,83,84].

Table 1 summarizes the methods and analytical characteristics of some selected configurations of electrochemical affinity biosensors using SPEs modified by grafting with aryldiazonium salts, which have been applied to clinical and food samples. In the following sections, we discuss the relevant aspects of such designs. 

### 2.1. Electrochemical Immunosensors Involving Aryl Diazonium Salt Chemistry onto Screen-Printed Electrodes

**Figure 2:**
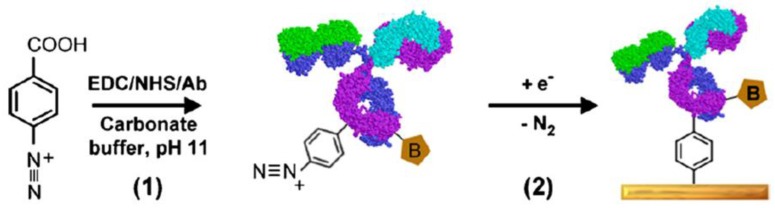
Preparation of a diazonium-modified antibody electrode: carboxyl diazonium is covalently attached to antibody by EDC/NHS (1) and diazonium–antibody is deposited onto an electrode by cyclic voltammetry (2). Reprinted and adapted from [26] with permission.

Strategies involving conjugation of capture antibodies with 4-carboxy methylaniline followed by diazotization to the respective diazonium salt modified antibody [23,24] (Figure 2), or with 4-carboxyphenyldiazonium salt to avoid the diazotization step in the presence of the protein, have been reported. Due to the high number of primary amines on the exterior, the proteins are indeed modified with several diazonium species.

More conventional strategies involve antibody immobilization onto electrodes previously modified with the diazonium salt. These strategies include: (i) modification of SPCEs [5,58,68,69,70,85], carbon nanomaterial-modified SPEs [33], graphene-modified SPEs (GSPEs) [59,60], carbon nanofiber-modified SPCEs (CNF SPCEs) [61] and reduced graphene oxide (rGO)-SPCEs [62,63] with 4-carboxyphenyl diazonium salt; (ii) modification of GSPEs with *p*-nitroaniline diazonium salt [20]; (iii) modification of graphite-based SPEs with *p-*aminophenyl acetic acid [58]; (iv) modification of glassy carbon electrodes (GCEs) with a mixed layer of oligo(ethylene glycol) species (OEG) and oligo(phenylethynylene) molecular wires (MW) [44].

In these immunosensing approaches, antibodies were covalently immobilized onto the electrografted surface using carbodiimide chemistry [5,33,58,59,60,61,62,63], glutaraldehyde [20], via an amidic bond [20] and through aminophenylboronic acid [85] or biotin-streptavidin interaction [44].

Electrochemical immunosensors have gained prominence for protein detection in recent years due to their sensitivity, selectivity, portability, simplicity, low cost, fast response time and compatibility with multiplexed determination and miniaturization [2,33,61]. The key points for the design of an electrochemical immunosensor are the choice of the electrode support and the immobilization of the antibody or the analyte onto its surface. As the following examples show, the chemistry of diazonium salts offers a very interesting alternative for the manufacture of immunosensors onto SPEs with attractive performance.

**Figure 3:**
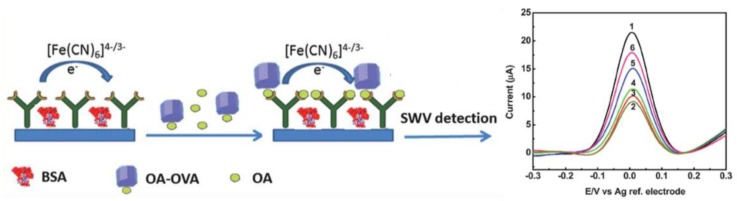
Schematic display of the working principle of the immunosensor based on a direct competitive format and SWVs of the immunosensor before the competition step (1) and after incubation with different concentrations of OA: 0.00 (2), 1.00 (3), 10.0 (4), 100 (5) and 1000 (6) ng L^−1^. Reprinted and adapted from [59] with permission.

A label-free voltammetric immunosensor for the detection of β-lactoglobulin was developed by electrochemical modification of GSPEs with 4-nitrophenyldiazonium cations. The diazonium cations were prepared by the diazotization of 4-nitroaniline. Subsequently, the in situ generated diazonium cations were covalently grafted through an electrochemical reduction step on the graphene electrode surface, followed by the electrochemical reduction of the terminal nitro to amine groups used, upon their activation with glutaraldehyde for covalent immobilization of β-lactoglobulin antibodies [20]. By monitoring the decrease in the DPV reduction peak current of [Fe(CN)_6_]^3−/4−^ in the presence of β-lactoglobulin, the immunosensor enabled a dynamic range from 1 pg mL^−1^ to 100 ng mL^−1^ and a LOD of 0.85 pg mL^−1^. Moreover, the immunosensor was applied to the analysis of different samples (cake, cheese snacks and sweet biscuits). Same authors proposed the use of the same GSPEs modified by cyclic voltammetry reduction of in situ generated 4-carboxyphenyldiazonium salt to develop voltammetric immunosensors for the sensitive detection of okadaic acid (OA) [59] and ovalbumin (OVA) [60]. The antibody was covalently immobilized onto the electrografted GSPE via carbodiimide chemistry. The immunosensor for OA determination was based on a competitive assay between OA and a fixed concentration of OA–ovalbumin conjugate (OA–OVA) for the immobilized antibodies and on measuring the [Fe(CN)_6_]^3−/4−^ reduction peak current obtained by SWV (Figure 3). The higher the concentration of OA, the larger the SWV signal due to the lower amount of immobilized OA-OVA. The method allowed reaching a LOD of 19 ng L^−1^, a linear determination range up to ~5000 ng L^−1^ and successful applicability for the analysis of spiked shellfish tissue extracts and certified reference mussel samples. The method developed for OVA, based on the decrease in the reduction peak of [Fe(CN)_6_]^3−/4−^ measured by DPV in the presence of the antigen, demonstrated to be appropriate for OVA determination in the concentration range from 1 pg mL^−1^ to 0.5 μg mL^−1^ with a LOD of 0.83 pg mL^−1^ and for the analysis of spiked cake extracts. 

Hayat et al. [5] developed another electrochemical immunosensor for OA through its covalent immobilization onto SPCEs modified by grafting with 4-carboxyphenyl film followed by terminal carboxylic group activation by *N*-hydroxysuccinimide (NHS) and *N*-(3-dimethylamino propyl)-*N*-ethylcarbodiimide hydrochloride (EDC). Hexamethyldiamine was then covalently bound by one of its terminal amine group to the activated carboxylic group. The carboxyl group of OA, activated by EDC/NHS, was then conjugated to the second terminal amine group on other side of the hexamethyldiamine through amide bond formation. After immobilization of OA, an indirect competitive immunoassay involving the labeling of a specific OA antibody with an alkaline phosphatase (AP)-labeled secondary antibody and DPV detection in the presence of 1-naphtyl phosphate (1-NP) was employed to detect OA. This immunosensor allowed a LOD of 1.44 ng L^−1^ and showed applicability to the analysis of certified reference mussel samples. One year later, same authors developed an impedimetric immunosensor for OA by covalent immobilization of the specific antibody onto the same carboxyphenyl-modified SPCE [58]. The increase in electron transfer resistance measured by electrochemical impedance spectroscopy (EIS) in the presence of [Fe(CN)_6_]^4−/3−^ was linearly proportional to the OA concentration in the 0.195–12.5 μg L^−1^ range, with a LOD of 0.3 μg L^−1^. The analysis performed in spiked mussel samples demonstrated acceptable recovery percentages.

**Figure 4:**
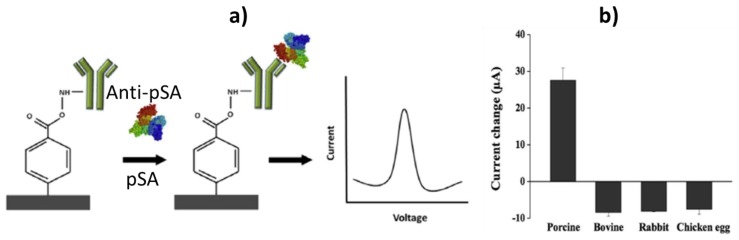
Electrochemical immunosensor developed for pSA determination onto a CNF SPE modified with a 4-carboxyphenyl layer (**a**) and comparison of the DPV responses provided by the immunosensor to 100 pg mL^−1^ of serum albumin from porcine, bovine, rabbit and albumin in chicken egg (**b**). Reprinted and adapted from [61] with permission.

A label-free electrochemical immunosensor for the detection of porcine serum albumin (pSA) using CNF SPEs with a 4-carboxyphenyl layer electrografted using the 4-carboxyphenyl diazonium salt was proposed by Lim et al. (Figure 4) [61]. Antibodies were covalently immobilized onto the modified electrodes previously activated with EDC/NHS and the increase in the SWV cathodic peak current of anionic redox probe recorded after immunocomplex formation with antibodies, attributed to the strong affinities of serum albumins like pSA towards anions, was used for the detection. This immunosensor exhibited a wide linear range (0.5–500 pg mL^−1^) and a low LOD (0.5 pg mL^−1^) in buffer solution. Moreover, the feasibility of this approach for practical application was demonstrated by satisfactory recoveries in spiked undiluted fresh raw pork samples.

Pingarrón´s group proposed the development of immunosensing platforms using SPCEs for the covalent immobilization of capture antibodies through a 4-ABA diazonium salt grafting strategy. Using this strategy, an electrochemical immunosensor for the determination of adrenocorticotropin hormone (ACTH) was reported [19]. The immunoelectrode design involved grafting with 4-ABA followed by using of amino phenylboronic acid for the oriented immobilization of anti-ACTH antibodies onto SPCE-modified electrode surfaces. A competitive immunoassay between the antigen and the biotinylated hormone for the binding sites of the immobilized antibody was performed. The electroanalytical response was generated by using alkaline phosphatase-labelled streptavidin and 1-naphtyl phosphate as the enzyme substrate. The electrochemical oxidation of the enzyme reaction product, 1-naphtol, measured by DPV was employed to monitor the affinity reaction. Under the optimized working conditions, an extremely low detection limit of 18 pg L^−1^ was obtained and an excellent selectivity against other hormones (cortisol, estradiol, testosterone, progesterone, hGH and prolactin) were achieved. The immunosensor was used to analyze a human serum sample containing a certified amount of ACTH with good results. This strategy was further extended to the construction of a novel dual electrochemical immunosensor for the multiplexed determination of adrenocorticotropin (ACTH) and cortisol onto dual screen-printed carbon electrodes (SPdCEs) [85].

**Figure 5:**
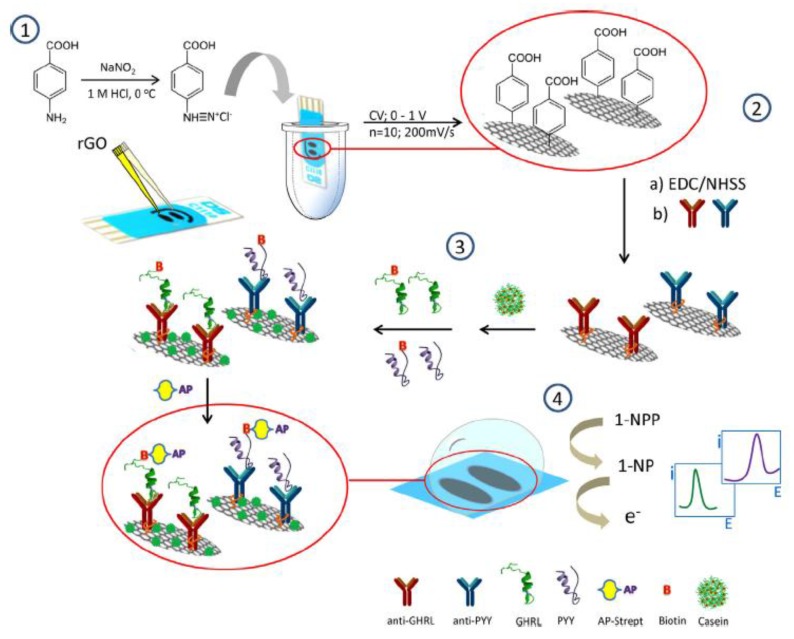
Schematic illustration of the different steps and protocols involved in the preparation and functioning of the dual GHRL and PYY immunosensor. Reprinted from [63] with permission.

Another configuration of a disposable immunosensing platform for the simultaneous determination of two obesity-related hormones, ghrelin (GHRL) and peptide YY (PYY) was also reported by this group [62,63]. SPdCEs were modified with reduced graphene oxide (rGO) and, after grafting of the diazonium salt of 4-ABA on these modified electrode surfaces, the corresponding capture antibody for each target hormone was covalently immobilized onto the 4-carboxyphenyl moieties of each working electrode via EDC/NHS chemistry (Figure 5). The determination of each hormone was performed by direct competitive immunoassays with the corresponding biotinylated hormones for the immobilized capture antibody. After labeling the attached biotinylated hormones with a polymer of streptavidin-phosphatase (AP-Strep), the DPV signal obtained in the presence of 1-NP was used to monitor the affinity reactions. This dual immunosensing scaffold provided linear current vs. log [hormone] plots extending between 10^−3^ and 100 ng mL^−1^ and 10^−4^ and 10 ng mL^−1^ for GHRL and PYY, respectively. The usefulness of this approach was also demonstrated by its application for the accurate determination of the analytes in spiked human serum and saliva samples.

**Figure 6:**
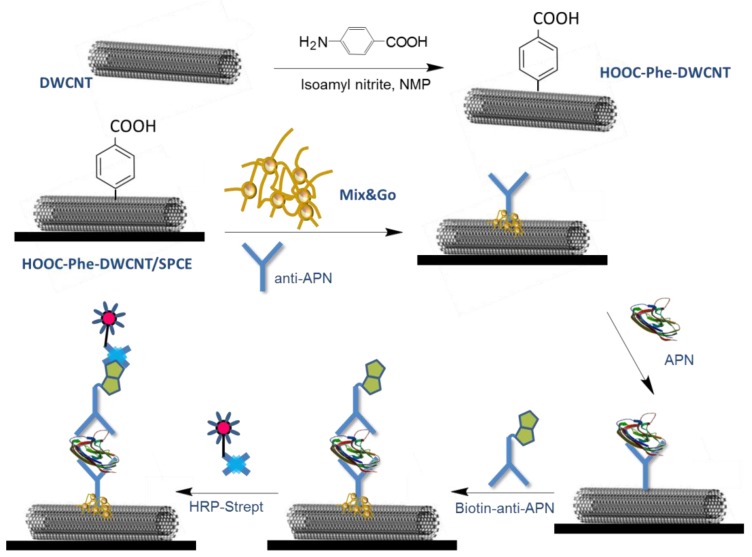
Schematic display of the different steps involved in the construction of an amperometric immunosensor for APN involving grafted DWCNTs and oriented immobilization of anti-APN by using the metallic-complex chelating polymer Mix & Go. Reprinted from [86] with permission.

SPCEs modified with double-walled carbon nanotubes (DWCNTs) previously functionalized with an aryl diazonium salt by the Bahr and Tour’ method [96] were used for developing an electrochemical immunosensor for adiponectin (APN). DWCNTs were treated with p-ABA in the presence of isoamyl nitrite in *N*-methyl-2-pyrrolidone (NMP) resulting in the formation of 4-carboxyphenyl-DWCNTs without any electrochemical treatment. The oriented binding of specific antibodies toward APN was accomplished by using the metallic-complex chelating polymer Mix&Go^TM^ (Figure 6). A calibration plot for APN was constructed with a range of linearity extending between 0.05 and 10.0 μg mL^−1^, and a detection limit of 14.5 ng mL^−1^. The usefulness of the immunosensor for the analysis of real samples was demonstrated by analyzing human serum from female or male healthy individuals [86].

A simple label-free impedimetric immunosensor for determination of mucin 4 (MUC 4) protein was proposed using graphite SPEs modified with *p-*aminophenylacetic acid for antibody immobilization via amidic bond [58]. The immunosensor provided a LOD of 330 pg μL^−1^. Eissa et al. [33] carried out a comparative study of six different carbon nanomaterial-modified electrodes (carbon, graphene (G), GO, single wall carbon nanotube (SWCNT), multi-wall carbon nanotube (MWCNT), and carbon nanofiber (CNF)) which were modified by grafting with 4-carboxyphenyl layers to develop voltammetric immunosensors for the detection of survival motor neuron (SMN) protein. The SMN antibody was covalently immobilized onto the terminal carboxylic moieties on the electrode surfaces through EDC/NHS chemistry. Results showed that the CNF-modified electrode exhibited the best performance for the SMN immunosensor. By measuring the increase in the SWV reduction peak current of [Fe(CN)_6_]^3−/4−^ in the presence of SMN due to the positive charge of the protein, the CNF-based immunosensor provided a LOD of 0.75 pg mL^−1^ and feasibility to perform the determination in a spiked blood sample from healthy volunteer. 

As it is well known, strategies allowing a high control over the fabrication of sensing interfaces on gold electrodes include mainly the use of SAMs [97,98] and, more recently, also modification with aryldiazonium salts [72]. This latter method provides not only an effective method for immobilization of bioreagents but also the stable incorporation of gold nanoparticles onto gold electrodes [82,83]. Although alkanethiols assembling offers a simply and versatile way to prepare sensing interfaces, their stability is a big issue when the as prepared sensors should operate over long measurement times or their fabrication involves multiple coupling steps. In these cases, the oxidation of alkanethiols and the resultant loss of SAM becomes problematical. In contrast to thiol–Au chemistry, diazonium modification on Au surfaces improves stability in terms of long-term storage in air, potential cycling under acidic conditions, and wider potential window for electrochemical detection methods [24,26]. Various examples of application of this methodology to gold electrode surfaces have been reported in the literature. For example, the direct modification of a gold electrode with aminophenyl groups by electrochemical reduction of in situ generated aminophenyl monodiazonium cations synthesized from *p-*phenylendiamine and NaNO_2_ was reported by Lyskawa and Bélanger [99]. Very recently, Phal et al. [100] prepared a gold electrode electrografted with 4-carboxybenzenediazonium for developing a methotrexate (MTX) immunosensor. However, only one work has been found regarding the use of screen printed gold electrodes [89]. In this method, SPAuE was modified with 4-nitrophenyl groups assembled from 4-nitrophenyl diazonium salt and, then, the nitro groups were electrochemically reduced to amines followed by activation with glutaraldehyde for covalently bind ochratoxin A (OTA) antibodies. A direct competitive-type immunosensor using OTA-HRP was prepared using 3,3′,5,5′-tetramethyl-benzidine (TMB) for the amperometric detection of OTA in a dynamic range up to 60 ng mL^−1^ with a LOD of 12 ng mL^−1^. 

### 2.2. Electrochemical Nucleic Acid Biosensors Involving Aryl Diazonium Salt Chemistry onto Screen-Printed Electrodes

Nucleic acid biosensors typically involve the immobilization of a single strand (ss) of a nucleic acid, typically a short oligonucleotide, to detect a complementary strand [101] or nucleic acid duplexes to detect small molecules, such as drugs [102] and of aptamers for a wide variety of (bio)molecules determination [21,69,70]. The immobilization of capture probes on electrode surfaces is thus a crucial step to obtain reliable nucleic acid biosensors [103]. A very attractive route involves covalent attachment of thiol-functionalized oligonucleotides on gold electrodes [104]. Regarding carbon electrode surfaces, although adsorption is one of the simplest techniques to immobilize nucleic acids [105,106,107,108], the resulting multiple-point linkage usually leads to poor hybridization efficiency and the release of the nucleic acids from the surface during the hybridization is a potential problem. To overcome these drawbacks, the covalent attachment of an oligonucleotide monolayer on a chemically-functionalized carbon surface appears as more advantageous because it allows increasing the sensitivity of the assay and the use of more stringent washing conditions to reduce the background signal. A very attractive method to covalently attach oligonucleotides on carbon surfaces relies on surface functionalization with diazonium groups [109,110]. During the coupling reaction, the amine electron-releasing group contained in the aromatic rings of adenine, guanine and cytosine undergo an electrophilic attack by in situ generated diazonium ions [65]. Moreover, as DNA sensors sometimes require reasonably high temperatures to ensure efficient duplexes denaturation or hybridization, the thermal stability of the linkage of the DNA to a substrate using diazonium salts chemistry is especially attractive [76]. The selected examples described below demonstrate the potential of this chemistry for developing electrochemical nucleic acid sensors with very attractive characteristics.

The novelty of diazonium chemistry towards the electroaddressable selective functionalization of a single electrode in an electrode array [24] was successfully explored to develop arrays for multi-determination of different proteins [26,111]. Moreover, dual functionality sensors able to detect simultaneously target DNAs and proteins were proposed also using this chemistry [24,25]. These approaches relied on modification of the electrodes in the array with 4-carboxyphenyl-diazonium salts further activated with EDC/NHS to allow the covalent attachment of biomolecules [24] and on the biomolecules modification with aryl diazonium prior to the addressing [25].

The first example of using aryldiazonium salt-derived layers for the preparation of nucleic acid-based biosensors involved the attachment of the DNA probe onto SPCEs modified with *p*-aminophenyl using *p*-nitroanilinodiazonium salt and further converted to diazophenyl functions [65]. Moreover, to reduce nonspecific adsorptions, the interface was modified with a mixed layer by spacing the nitrophenyl moieties using carboxyphenyl molecules. Other binary films of 4-((tri-methylsilyl)ethynyl) benzene (TMSi-Eth-Ar) and *p*-nitrobenzene (*p*-NO_2_-Ar) prepared sequentially by electrografting onto SPCEs were used after deprotection and in the presence of copper(I) catalyst to attach aptamers with azide function by forming a covalent 1,2,3-triazole linkage [21].

Covalent immobilization of amino terminated aptameric [69,70] and PNA [68] probes have been successfully achieved using carbodiimide chemistry onto SPCEs modified via diazonium coupling reaction with 4-aminobenzoic acid. A different DNA immobilization strategy relied on the selective covalent binding between thiols and maleimides [112] and the coupling of the 4-phenylmaleimide diazonium salt to a thiol-terminated oligonucleotide prior surface assembly. Another novel strategy for immobilizing DNA and preparing aryldiazonium salts [113] involved the use of a triazene (a protected aryldiazonium salt) and its activation using dimethyl sulfate to reveal the diazonium moiety. In the DNA electrochemical sensor reported, a unique ferrocene derivative (used as electrochemical reporter) was prepared with a phenyl triazene attached to one of the ferrocene cyclopentadienyl (Cp) ring and a succinimide ester to the other Cp ring. An aminated DNA was further attached to this activated ester.

DNA-sensing platforms for the determination of an amplified herpes virus DNA sequence were prepared by attaching covalently oligonucleotide capture probes onto *p*-aminophenyl functionalized SPCEs using *p*-nitroanilinodiazonium salt [65]. The subsequent conversion of the *p-*aminophenyl groups to diazophenyl moieties provided a convenient and versatile way to covalently link nucleic acids on carbon surfaces. In this approach, the PCR-amplified 406 base-pairs (bp) human cytomegalovirus (HCMV) DNA sequences were detected through a sandwich-type hybridization assay with the immobilized DNA probe and a colloidal gold-labeled detector probe. The hybridization event was followed by measuring the Au(III) ions generated by acid dissolution of the gold metal nanoparticles attached to the hybrids using anodic stripping voltammetry at a screen-printed microband electrode (SPMBE).

**Figure 7:**
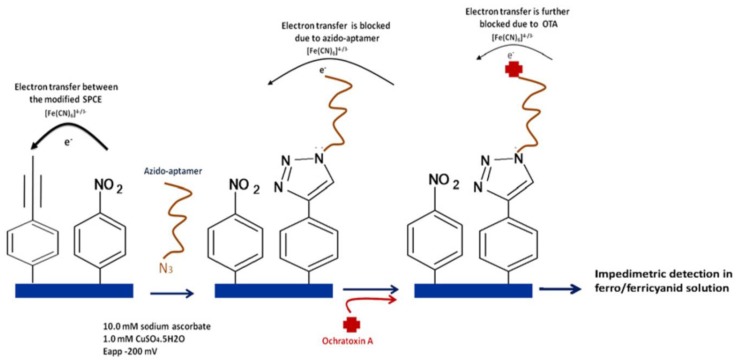
Schematic display of the working principle of the impedimetric aptasensor for the determination of OTA. Reprinted from [21] with permission.

Hayat et al. [21] developed a highly sensitive and reusable aptasensor for the impedimetric detection of ochratoxin A (OTA) by covalent immobilization of a specific aptamer onto SPCEs modified with two binary films of diazonium salts via click chemistry. SPCEs were modified sequentially by electrografting of protected layers of 4-((trimethylsilyl)ethynyl) benzene (TMSi-Eth-Ar) and *p*-nitrobenzene (p-NO_2_-Ar) by means of electrochemical reduction of their corresponding diazonium salts. After deprotection, by treatment with tetrabutylammonium fluoride (TBAF), and in the presence of copper (I) catalyst, the active ethynyl groups of the film and the azide moiety of the aptamer reacted efficiently forming a covalent 1,2,3-triazole linkage (Figure 7). The increase in the electron-transfer resistance (R_CT_) values measured by EIS in the presence of [Fe(CN)_6_]^4−/3−^ was proportional to the OTA concentration between 1.25 and 500 ng L^−1^ with a LOD of 0.25 ng L^−1^. The aptasensor could be regenerated 10 times with a mild solution, showed a storage stability of at least 10 days at 4 °C, and was successfully applied to the analysis of spiked beer samples.

**Figure 8:**
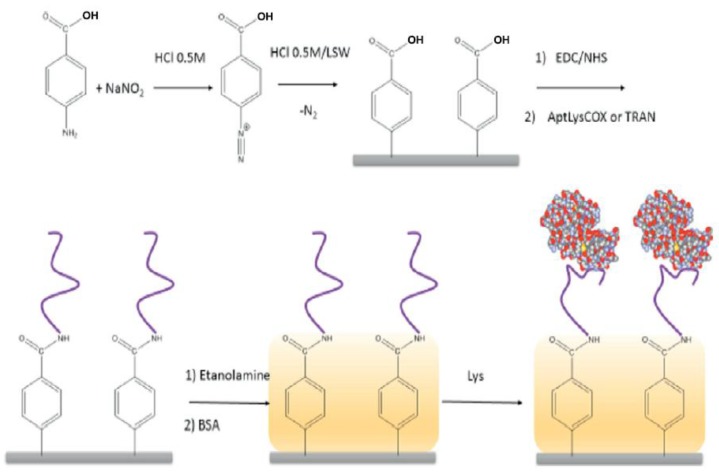
Impedimetric aptasensor developed for Lys determination on a 4-ABA-modified SPCE. Reprinted from [69] with permission.

The use of an aptamer immobilized on SPCEs through diazonium coupling reaction using 4-ABA was also exploited by Del Valle’s group to develop competitive aptasensors for lysozyme (Lys) determination. One of these approaches involved a direct assay and impedimetric detection providing linearity between 0.025 and 0.8 mM, and a LOD of 25 nM (Figure 8) [69]. Another reported method relied on an aptamer-antibody sandwich assay involving the use of a biotinylated antibody further labeled with avidin-AP. By DPV monitoring of the oxidation signal of 1-naphthol generated by enzymatic hydrolysis of 1-naphthyl phosphate substrate, the method exhibited a wide detection range (5 fM–5 nM) and a LOD 4.3 fM [70]. Both aptasensors were used for the quantification of the target protein in spiked wine samples. Additional claimed advantages of these aptasensors are the use of simple instrumentation, low production cost and rapid response.

SPCEs fabricated using Low Temperature Co-fired Ceramics (LTCC) technology and functionalized with amino-modified PNA probes by electrografting via in situ generated diazonium cations (4-ABA) were proposed for label-free molecular detection of antibiotic resistance by targeting *bla_NDM_*, one of the main antimicrobial resistance factors in carbapenem-resistant Enterobacteriaceae. This impedimetric PNA-based biosensor provided a LOD of 200 nM [68]. The excellent molecular detection performance combined with a low cost and accelerated sensor manufacturing and functionalization process (over six times faster than protocols used for gold electrodes) make this platform very attractive for rapid detection of antimicrobial resistance at *point-of-care* POC with competitive costs.

### 2.3. Other Electrochemical Biosensors Involving Aryl Diazonium Salt Chemistry onto Screen-Printed Electrodes

**Figure 9:**
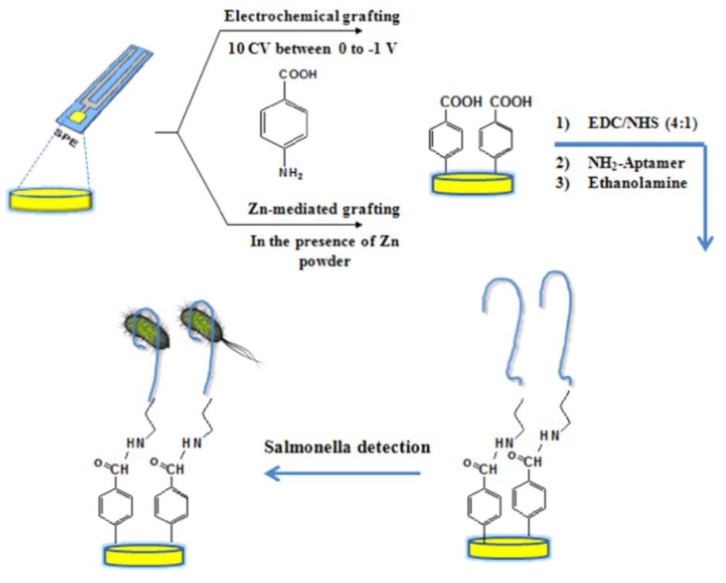
Overview of the preparation of the *S. typhimurium* aptasensor. Reprinted from [95] with permission.

Apart from antibodies and nucleic acids, aryl diazonium salt derived layers have also been used for cells immobilization [74,114]. For this purpose, a novel phenyl boronic acid pinacol ester diazonium salt was synthesized so that, after assembly onto the electrode surface, addition of sodium iodate removed the pinacol protecting group to yield a phenylboronic acid functionalized surface. By exploiting the selective reaction of boronic acid with sugars, these scaffolds were used to immobilize yeast cells [74] and murine macrophages (mammalian cells belonging to the immune system) [114]. Claimed advantages of these bioscaffolds include the possibility to release the captured cells from the surface by exposure to fructose due to the competitive reaction of this sugar with the cells for the boronic acid of the functionalized surface. Furthermore, a label free impedimetric aptamer sensor for *Salmonella typhimurium* (*S. typhimurium*) detection was reported by Bagheryan et al. [95]. A diazonium-supporting layer was fabricated onto a SPCE followed by immobilization of aminated aptamer (Figure 9). The developed design responded linearly over the 10–10^8^ CFU mL^−1^ range with a limit of detection of 6 CFU mL^−1^. Good recoveries were obtained in the application to spiked apple juice.

## 3. General Considerations, Challenges and Prospects

Electrochemical grafting consisting of covalent modification of carbon surfaces by aryl radicals generated from electrochemical reduction of diazonium salts has demonstrated to facilitate the electron transfer and provide a highly stable binding surface with enhanced properties for selective and controlled immobilization of chemical and biological compounds [62,64]. Advantages of this method include the ease of diazonium preparation, a covalent attachment to the electrode, the speed of the chemistry involved [29] and the ability to modify closely-spaced electrodes with different biological entities (proteins, nucleic acid strands, and peptides) [23,29]. Moreover, the application of aryldiazonium salts for (bio)sensing has recently seen the integration of nanomaterials together with the recognition species into interfaces thus taking advantage of the nanomaterials properties. 

As it has been pointed out above, diazonium chemistry-based biosensing platforms include SPCEs [5,58,68,69,70,85], carbon nanomaterial-modified SPEs [33], GrSPEs [59,60], CNF SPEs [55], rGO-SPCEs [62,63] and graphite-based SPEs [64]. Regarding the aryl diazonium substrates, 4-ABA [5,33,58,59,60,61,62,63,68,69,70,85], *p*-nitroaniline [20] and *p-*aminophenylacetic acid [64] were mostly used. 

Despite the modification of surfaces using aryl diazonium salts has been explored for almost 20 years now, the application of aryl diazonium salts for fabricating biosensing interfaces is still in its infancy. Although variations of aryldiazonium salts mixed layers have been employed for affinity biosensing at conventional electrodes [30], they have been scarcely explored onto disposable electrodes. Therefore, additional efforts should be focused on exploring multicomponent layers and to prepare more stable analogues of sensing interfaces that those developed with another surface chemistry.

However, it is worth to mention that despite these issues, the exciting advances made in the understanding of aryl diazonium salt chemistry, and how to assemble complex layers on surfaces, the range of new synthesized aryldiazonium salts, and the incorporation of nanomaterials are providing aryl diazonium salts derived sensors with the stability and flexibility advantages this chemistry provides. Furthermore, possible drawbacks, relative to other surface chemistries such as that of the alkanethiols, that are in a much more advanced state, are rapidly being resolved which makes aryldiazonium salt chemistry almost the ideal surface chemistry for preparing sensing interfaces.

## Figures and Tables

**Table 1:** Electrochemical affinity biosensors involving aryldiazonium salt chemistry onto screen printed electrodes.

Electrode	Configuration	Analyte	Detection Scheme	Detection Technique	Linear Range	LOD	Sample	Ref.
SPCE	Sandwich immunosensor, grafted *p-*ABA-4-APBA-anti-ACTH-ACTH-Biotin-Strept-AP	ACTH	1-NPP + AP → 1-NP 1-NP → quinone	DPV	0.025–1.0 pg mL^−1^	18 pg L^−1^	serum	[19]
SPdCE	Sandwich immunosensor, grafted *p-*ABA-4-APBA-anti-ACTH-ACTH-Biotin-Strept-AP Direct competitive, grafted *p-*ABA-4-APBA-anti-cortisol-cortisol-AP	ACTH cortisol	1-NPP + AP → 1-NP 1-NP → quinone	DPV	5.0 × 10^−5^–0.1ng mL^−1^ 0.1–500 ng mL^−1^	40 pg L^−1^ 37 pg mL^−1^	serum	[85]
SPCE	Sandwich immunosensor, grafted *p-*aminothio-phenol-AuNPs-anti-BNP-BNP-anti-BNP-HRP	BNP	H_2_O_2_ + HRP + HQ → quinone	amperometry	0.014–15 ng mL^−1^	4 pg L^−1^	serum	[84]
SPCE	Sandwich immunosensor, grafted *p*-ABA-DWCNTs-M&G-anti-APN-APN-Biotin-Strept-HRP	APN	H_2_O_2_ + HRP + HQ → quinone	amperometry	0.05–10.0 μg mL^−1^	14.5 ng mL^−1^	serum	[86]
SPdCE	Direct competitive immunosensor, grafted *p*-ABA-anti-GHRL GHRL-Biotin-Strept-HRPDirect competitive, grafted *p*-ABA-anti-PYY- PYY-Biotin-Strept-HRP	GHRLPYY	H_2_O_2_ + HRP + HQ → quinone	DPV	10^−3^–100 ng mL^−1^ 10^−4^–10 ng mL^−1^	1.0 pg mL^−1^ 0.02 pg mL^−1^	serum, saliva	[62]
SPdCE	Sandwich immunosensor, grafted *p*-ABA-DWCNTs -M&G-anti-TNF-TNF-anti-TNF-Biotin-poly-Strept-HRP. Sandwich, grafted *p*-ABA-DWCNTs-M&G-anti-IL1β-IL1β-anti-IL1β-Biotin-poly-Strept-HRP	TNF-α IL-1β	H_2_O_2_ + HRP + HQ → quinone	amperometry	1–200 pg mL^−1^ 0.5–100 pg mL^−1^	0.85 pg mL^−1^ 0.38 pg mL^−1^	serum saliva	[87]
SPCE	Sandwich immunosensor, grafted *p*-ABA-Strept-Biotin-anti-TGF-TGF-anti-TGF-SWCNT(-HRP)-Phe-V	TGF-β1	H_2_O_2_ + HRP + HQ → quinone	amperometry	2.5–1000 pg mL^−1^	0.95 pg mL^−1^	saliva	[88]
SPAuE	Direct competitive immunosensor, grafted 4-nitrophenyl red.-GA-anti-OTA-HRP	OTA	TMB reduction	amperometry	up to 60 ng mL^−1^	12 ng mL^−1^	-	[89]
SPCE	Indirect competitive immunosensor, grafted 4-carboxy-phenyl-HMDA-OA-anti-OA-IgG-AP	OA	1-NPP + AP → 1-NP 1-NP → quinone	DPV	-	1.44 ng L^−1^	mussel	[5]
SPCE array	Sandwich immunosensor, grafted 4-aminophenyl-GA-anti-CEA(or anti-AFP)-anti-CEA(or anti-AFP)-HRP	CEAAFP	H_2_O_2_ + HRP + HQ → quinone	amperometry	0.10–50 ng mL^−1^	0.03 ng mL^−1^ 0.05 ng mL^−1^	serum	[90]
SPCE	Label free immunosensor, grafted 4-carboxyphenyl-anti-OA-OA	OA	R_CT_ [Fe(CN)_6_]^4−/3−^	EIS	0.195–12.5 μg mL^−1^	0.3 μg L^−1^	mussel	[58]
GrSPE	Label free direct competitive immunosensor, grafted 4-carboxyphenyl-anti-OA-OA-OVA	OA	reduction current [Fe(CN)_6_]^4−/3−^	SWV	up to 5000 ng mL^−1^	19 ng L^−1^	shellfish tissue	[59]
GrSPE	Label free immunosensor, grafted 4-nitrophenyl red.-GA-anti-β-LGB-β-LGB	β-LGB	reduction current [Fe(CN)_6_]^4−/3−^	DPV	1 pg mL^−1^–100 ng mL^−1^	0.85 pg mL^−1^	cake, cheese, snacks	[20]
CnFSPE	Label free immunosensor, grafted 4-carboxyphenyl-anti-pSA-pSA	pSA	reduction current [Fe(CN)_6_]^4−/3−^	DPV	0.5–500 pg mL^−1^	0.5 pg mL^−1^	fresh meat	[61]
CnFSPE	Label free immunosensor, grafted 4-carboxyphenyl-anti-pSA-pSA	SMN	reduction current [Fe(CN)_6_]^4−/3−^	SWV	1.0 pg mL^−1^–100 ng mL^−1^	0.75 pg mL^−1^	whole blood	[33]
GrSPE	Label-free immunosensor, grafted 4-carboxyphenyl-anti-OVA-OVA	OVA	reduction current [Fe(CN)_6_]^4−/3−^	DPV	1.0 pg mL^−1^–0.5 μg mL^−1^	0.83 pg mL^−1^	egg-free cake	[60]
SPCE	Label free aptasensor, grafted 4-carboxyphenyl-aptamer AFB1	AFB1	R_CT_ [Fe(CN)_6_]^4−/3−^	EIS	0.125 ng mL^−1^–16 ng mL^−1^	0.12 ng mL^−1^	beer wine	[91]
SPCE	Label free immunosensor, grafted 4-carboxyphenyl-acetic-anti-MUC4	MUC4	R_CT_ [Fe(CN)_6_]^4−/3−^	EIS	1–15 μg mL^−1^	0.33 μg mL^−1^	serum	[64]
SPCE	Label free aptasensor, grafted *p*-ABA -aptamer-OTA	OTA	R_CT_ [Fe(CN)_6_]^4−/3−^	EIS	0.15–2.5 ng mL^−1^	0.15 ng mL^−1^	cocoa beans	[92]
SPCE	Label free aptasensor, grafted 4-carboxyphenyl-aptamer-AFM1	AFM1	R_CT_ [Fe(CN)_6_]^4−/3−^	EIS	2–150 ng L^−1^	1.15 ng L^−1^	milk	[93]
SPCE	Label free DNA sensor, grafted 4-nitrophenyl red.-DNA	*MRP3* gene	reduction current Ru(NH_3_)_6_^3+^	DPV	1.0–7.3 ng μL^−1^	210 pg μL^−1^	-	[94]
SPCE	Label free aptasensor, grafted 4-carboxyphenyl-NH_2_-aptamer-salmonella	*S. typhi.*	R_CT_ [Fe(CN)_6_]^4−/3−^	EIS	10–10^8^ CFU mL^−1^	6 CFU mL^−1^	apple juice	[95]
SPCE	Sandwich aptasensor, grafted 4-carboxyphenyl-aptamer-anti-Lys-Biotin-avidin-AP	Lys	1-NPP + AP → 1-NP 1-NP → quinone	DPV	5 fM–5 nM	4.3 fM	wine	[70]
SPCE	Label free aptasensor, grafted 4-((trimethylsilyl)ethynyl) benzene- *p*-nitrobenzene + click azide aptamer-OTA	OTA	R_CT_ [Fe(CN)_6_]^4−/3−^	EIS	1.25–500 ng L^−1^	0.25 ng L^−1^	beer	[21]

ACTH, adrenocorticotropin; 2-ABA, 2-aminobenzyl amine; *p*-ABA, *p*-aminobenzoic acid; AFB1, aflatoxin B1; AFM1, aflatoxin M1; AP, alkaline phosphatase; 4-APBA, 4-aminophenylboronic acid; APN, adiponectin; AuNP, gold nanoparticles; BNP, brain natriuretic peptide; CEA, carcinoembrionic antigen; CnFSPE, carbon nanofibers screen printed electrode; DPV, differential pulse voltammetry; DWCNTs, double walled carbon nanotubes; EIS, electrochemical impedance spectroscopy; GQDs, graphene quantum dots; GHRL, ghrelin; GrSPE, graphene-modified screen-printed carbon electrode; HQ, hydroquinone; HRP, horseradish peroxidase; IL-1β; interleukin-1β; β-LGB, β-lactoglobulina; Lys, lysozyme; M&G, Mix&Go;MRP3 gene; MUC4, mucin 4; 1-NP, 1-naphthylphenol; 1-NPP, 1-naphthylphosphate; OA, okadaic acid; OTA, ochratoxin A; OVA, ovoalbumin; PYY, peptide YY; pSA, porcine serum albumin; R_CT,_ charge transfer resistance; S. typhi., Salmonella typhimurium; SMN, survival motor neuron: SPAuE, screen printed gold electrode; SPCE, screen printed carbon electrode; SPdCE, screen printed dual carbon electrode; SWCNT, single walled carbon nanotubes; SWV, square wave voltammetry; TGF-β1, transforming growth factor β1; TNF-α, tumor necrosis factor alpha; V, viologen.
